# Motives for (not) participating in a lifestyle intervention trial

**DOI:** 10.1186/1471-2288-8-17

**Published:** 2008-04-10

**Authors:** Jeroen Lakerveld, Wilhelmina IJzelenberg, Maurits W van Tulder, Irene M Hellemans, Jan A Rauwerda, Albert C van Rossum, Jaap C Seidell

**Affiliations:** 1 Department of General Practice, EMGO Institute, VU University Medical Center, v.d. Boechorststraat 7, 1081 BT, Amsterdam, The Netherlands; 2 Institute of Health Sciences, Faculty of Earth and Life Sciences, VU University, Amsterdam, The Netherlands; 3 Department of Vascular Surgery, VU University Medical Center, Amsterdam, The Netherlands; 4 Department of Cardiology, VU University Medical Center, Amsterdam, The Netherlands

## Abstract

**Background:**

Non-participants can have a considerable influence on the external validity of a study. Therefore, we assessed the socio-demographic, health-related, and lifestyle behavioral differences between participants and non-participants in a comprehensive CVD lifestyle intervention trial, and explored the motives and barriers underlying the decision to participate or not.

**Methods:**

We collected data on participants (n = 50) and non-participants (n = 50) who were eligible for inclusion in a comprehensive CVD lifestyle interventional trial. Questionnaires and a hospital patient records database were used to assess socio-demographic, health-related and lifestyle behavioral variables. Univariate and multivariate logistic regression was used to describe the relationship between explanatory variables and study participation. Furthermore, motives and barriers that underlie study participation were investigated by means of questionnaires.

**Results:**

Participants were younger, single, had a higher level of education and were employed. No statistically significant differences were found in health measures and behavioral variables. The motives for participation that were most frequently reported were: the perception of being unhealthy and willingness to change their lifestyle. The main barriers reported by non-participants were financial arguments and time investment.

**Conclusion:**

The differences between participants and non-participants in a lifestyle intervention trial are in mainly demographic factors. The participants consent in order to alter their lifestyle, and/or because they want to improve their health. To minimize non-participation, it is recommended that access to a lifestyle intervention program should be easy and cause no financial restraints.

**Trial registration:**

ISRCTN69776211.

## Background

The challenge in the recruitment of patients for clinical trials is to minimize non-participation among the target population. Non-participants can have a considerable influence on the external validity of a study [1,2], and make it difficult to meet the goals of recruitment within a specified time-limit and budget [3]. Enrollment rates in clinical trials vary, and obtaining informed consent from patients may sometimes be difficult [4]. Reasons for non-participation that have been reported by patients with heart failure are the perception of being too unwell, lack of transport, being too old, or too busy [5]. Living too far away from the study site or indifference towards the study [6], as well as concerns about inconvenience/annoyance and the possibility of being randomized to the control group have also been reported as barriers to participation [7]. Motivations for participation seem to be altruistic perceptions and perceived benefits [7]. Earlier investigations of unwanted selective inclusion in trials involving cardiovascular disease (CVD) patients show that smokers [7], older patients [5,7,8], women [5,7], and patients who live further away from the study site [6] are sub-groups that are less likely to consent.

The mechanisms underlying participation and the threat for the external validity of the study results have scarcely been investigated in rehabilitation or secondary prevention programs for CVD patients. A non-participation survey among CVD patients who were offered an intensive rehabilitation program demonstrated that the participants were younger, still working and had a higher level of education than the non-participants [9]. At present, the emphasis of secondary prevention in CVD patients lies increasingly on multifactorial risk reduction through lifestyle intervention therapy [10,11], but the non-participation of CVD patients has not yet been evaluated in lifestyle intervention trials.

**Figure 1:**
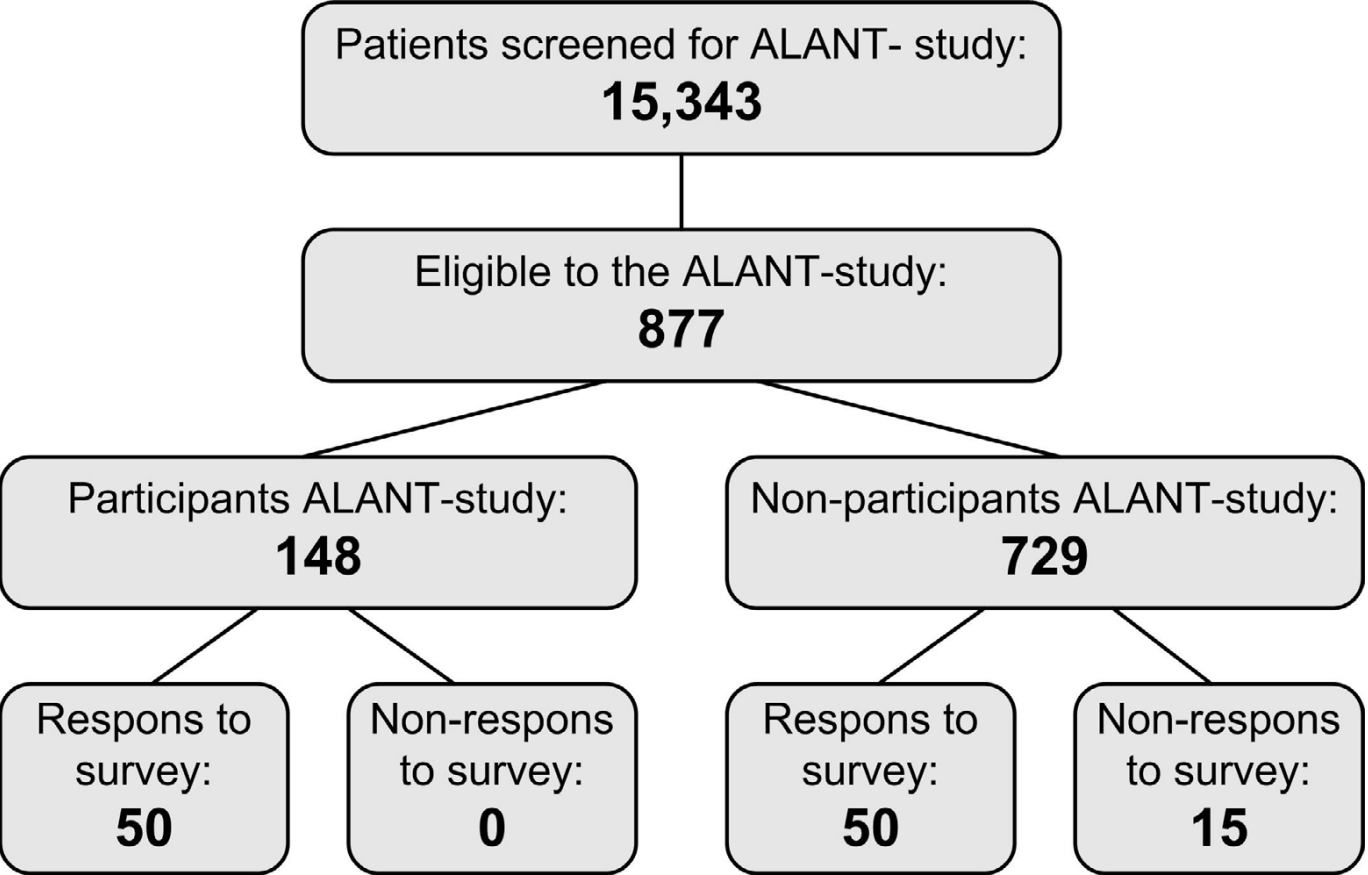
Flow chart of patient recruitment in the ALANT Study and in the non-participant survey.

At the VU University in Amsterdam we are conducting the ALANT study (Activity, Lifestyle And Nutritional Therapy-study) on the cost-effectiveness of a multifactorial comprehensive lifestyle intervention program for CVD patients. The program has already been described in more detail in an intervention study with no control group [12]. Figure 1 is a flow chart of patient recruitment for the ALANT study and the present survey. A total of 15,343 patients were screened for the ALANT study, 877 of whom were assessed by the medical specialists as eligible for participation in the study. The other patients did not meet the inclusion criteria, or did not show up for their appointment, or were not assessed by the medical specialists. A total of 729 (83%) of the 877 eligible patients decided not to participate. We therefore evaluated the socio-demographic, health-related, and lifestyle behavioral differences between participants and non-participants in this trial. Motivational aspects involved in trial participation and barriers for participation were also evaluated in order to enable us to make recommendations for successful patient recruitment in the future.

## Methods

### Participants

Eligible patients from the vascular and cardiology outpatient departments of the Academic Hospital of the VU University were invited to participate in the ALANT study by their medical specialist during consulting-hours. In addition, potentially eligible patients were identified in the databases of the departments of cardiology and vascular surgery. Members of the research team contacted these patients by telephone shortly after having sent them a letter of invitation by mail. Consecutive participants and non-participants in the ALANT study were then invited to take part in our survey, the protocol of which was reviewed and approved by the Medical Ethics Committee of the VU Medical Center.

Eligible for participation in the ALANT study were patients with stable CVD, between 18 and 75 years of age, with at least one of the following risk factors: smoking, diabetes, hypertension, dyslipidemia, hypercholesterolemia, overweight/obesitas or not meeting the recommended level of regular physical activity. Patients who were unable to climb a flight of stairs or to communicate adequately in the Dutch language were excluded from participation, as well as those who had unstable CVD or diabetes with a glycated hemoglobin (HbA_1c_) above 10.0%. Finally, their comorbidity had to be stable, and there should be no contra-indication for them to engage in physical exercise, as assessed by the referring physician. CVD was defined as coronary heart disease, a history of transient ischemic attack, cerebrovascular accident, or signs of peripheral vascular disease. The Body Mass Index (BMI) was calculated as weight (in kg)/height (in m)^2^, and was used to identify patients who were overweight (BMI >25) or obese (BMI >30). Hypertension was defined as the use of antihypertensive medication, or systolic blood pressure above 140-mmHg and/or diastolic blood pressure above 90 mmHg. The recommended level of physical activity was defined as moderate intensive exercise for at least half an hour, five times a week (i.e. brisk walking, cycling or other forms of brisk exercise).

Patients had to be referred by a physician in order to obtain compensation from their health insurance company, but this did not include a financial contribution of € 150 for patients allocated to the lifestyle program.

The inclusion of participants and non-participants in this survey was stopped when 50 questionnaires had been filled in and returned by each group. This cut-off point was chosen in order to achieve a representative, yet feasible sample in both groups.

### Measurements

Data were collected from questionnaires and the patient record database of the VU Medical Center. Successive non-participants in the ALANT study were asked by telephone to complete and return a questionnaire, and two gift vouchers were raffled among the non-participants who did so. Patients who did not return the questionnaire after two weeks received another questionnaire by mail and a booster telephone call shortly afterwards. Successive participants in the ALANT study were asked to fill in the same questionnaire during their first measurement visit.

The following socio-demographic, health-related and behavioral variables were studied:

### Socio-demographic variables

Gender, age, marital status (married/living together, yes/no), level of educational (none or primary education, secondary education, higher education), job status (working, unemployed, retired), distance from the patient's home to the hospital, and ethnicity. The term *ethnic minority *was used when at least one of the patient's parents had not been born in the Netherlands [13].

### Health-related variables

#### - Perceived general health

Perceived general health was assessed with one question derived from the SF-36 ('how is your health in general?') [14] and the EuroQol [15]. The EuroQol questionnaire assesses the general health status in 5 dimensions: mobility, self-care, usual activities, pain/discomfort, and anxiety/depression. Based on the model developed by Lamers et al., the total score was expressed in utilities (0–1) according to values for the general population of the Netherlands and subsequent statistical modeling [16].

#### - Clinical variables

BMI was calculated on the basis of self-reported weight and height. The variables *hypertension *and *co-morbidity diabetes *were retrieved from the patient's most recent medical records, dating from the previous two years.

### Behavioral variables

#### - Lifestyle behaviors

The questionnaire contained questions about current smoking, pack years (average number packs of 20 cigarettes per day smoked, multiplied by the number of years as a smoker), and meeting the recommended levels of physical activity (yes/no).

#### - Stages of behavioral change

Stages of behavioral change in physical activity were determined according to the trans theoretical model TTM [17]. The TTM identifies specific stages that individuals go through when trying to change their behavior. The stages of change include pre-contemplation (not thinking about making changes), contemplation (thinking about making changes, but not immediately), preparation (planning to make changes within the next 30 days, and may already be making small changes), action (initiated the changes in the past 6 months), and maintenance (maintained the changes for more than 6 months) [17].

### Motives and barriers for participation

A four-item Likert scale was used to assess the role of various determinants that were presumed by the investigators to influence the decision to participate or not (time investment, costs, interest in the study, changing daily routine, poor health status, health 'too good', and distance to study site/transport). The questionnaire also contained one open question: 'Why did you decide (not) to participate in the study?' Two members of the research team independently categorized the answers to this open question; disagreements were resolved after discussion.

### Statistical analysis

Socio-demographic and clinical group means were compared, using Chi-square tests for categorical data and independent T-tests for continuous data. Univariate logistic regression was used to describe the relationship between explanatory variables and study participation (yes/no). The multivariate model was then constructed with variables that had a univariate *P *value of 0.1 or less. The step-forward procedure was followed by first putting the variable with the lowest *P *value in the model, followed by the next lowest, and so on. Variables with a *P *value of less than 0.05 were retained in the model and the other variables were omitted, unless they changed the odds ratios (OR) of the independent variable or one of the other variables by more than 10%. The level of significance was set at *P *< 0.05, and the statistical analysis was performed in SPSS, version 12.0.1 [18].

## Results

All of the 65 non-participants who were contacted were reached by phone, and they all agreed to complete a questionnaire, but 15 (23%) of the non-participants did not return the questionnaire. All of the 50 participants who had been invited agreed to participate in this survey and completed the questionnaire.

### Socio-demographic, health-related, and lifestyle behavioral differences

The characteristics of the non-participants and the participants are presented in Table 1. There were statistical differences with regard to age, level of education and working situation. Marital status differed, but not statistically.

**Table 1:** Characteristics of participants and non-participants in a multifactorial comprehensive lifestyle intervention trial for patients with CVD

	**Participants (n = 50)**	**Non-participants (n = 50)**	**95% CI**
**Socio-demographic**			
			
Sex: male	35 (70.0%)	39 (78.0%)	-0.25 to 0.09
Age (mean ± SD)	60.2 ± 8.9	64.3 ± 8.3	0.72 to 7.53*
Married or living together	29 (60.0%)	38 (76.0%)	-0.33 to 0.03
Level of education			
- Low	15 (31.3%)	24 (48.0%)	-0.34 to 0.03
- Middle	15 (31.3%)	23 (46.0%)	-0.32 to 0.04
- High	18 (37.5%)	3 (6.0%)	0.16 to 0.46*
Job status			
- Retired	17 (35.4%)	30 (60.0%)	-0.42 to -0.05*
- Working	17 (35.4%)	6 (12.0%)	0.07 to 0.39*
- Unemployed	14 (29.2%)	14 (28.0%)	-0.16 to 0.19
Ethnic minority	10 (21.3%)	6 (12.5%)	-0.07 to 0.24
Distance to hospital in km (mean ± SD)	9.9 ± 7.4	12.4 ± 15.9	-2.43 to 7.42

**Health-related variables**			
			
*Perceived general health*			
SF36			
- Good, very good or excellent	26 (54.2%)	28 (56.0%)	-0.21 to 0.17
- Fair or poor	22 (45.8%)	22 (44.0%)	-0.17 to 0.21
EuroQol (mean ± SD)	0.73 ± 0.3	0.80 ± 0.2	-0.04 to 0.18
			
*Clinical*			
BMI (mean ± SD)	28.1 ± 5.4	27.0 ± 3.8	-2.99 to 0.73
Overweight (25<BMI < 30)	20 (40.0%)	23 (46.9%)	-0.25 to 0.12
Obese (BMI > 30)	16 (32.0%)	8 (16.3%)	-0.01 to 0.32
Co-morbidity diabetes	9 (18.0%)	10 (20.0%)	-0.17 to 0.14
Hypertension	29 (58.0%)	30 (60.0%)	-0.21 to 0.17

**Behavioral variables**			
			
*Lifestyle behaviors*			
Current smoker	17 (39.5%)	16 (32.0%)	-0.12 to 0.26
Pack years (mean ± SD)	19.5 ± 19.3	14.7 ± 16.9	-12.32 to 2.73
Not meeting recommended levels of physical activity	33 (66.0%)	32 (65.3%)	-0.27 to 0.33
			
*Stages of behavioral change*			
Pre-contemplation	2 (4.2%)	8 (16.3%)	-0.25 to 0.00
Contemplation	5 (10.4%)	5 (10.2%)	-0.13 to 0.13
Preparation	8 (16.7%)	4 (8.2%)	-0.05 to 0.22
Action	9 (18.8%)	12 (24.5%)	-0.22 to 0.11
Maintenance	24 (50.0%)	20 (40.8%)	-0.10 to 0.28

Statistical significance was assessed with Chi-square tests for categorical variables and t-tests for continuous variables.

CI: Confidence interval.

SD: Standard deviation.

BMI: Body Mass Index.

* *P *< 0.05

In the univariate logistic regression model, the variables age, high level of education, still working, and being retired showed a significant relationship with study participation. These variables remained statistically significant in the multivariate model (with the exception of being retired), but being single also appeared to be highly significantly associated with study participation (Table 2).

**Table 2:** The univariate and multivariate association of variables with participation in a multifactorial comprehensive lifestyle intervention trial for CVD patients

**Univariate**	**Odds Ratio (95% CI)**
Age: one year older	0.95 (0.90–0.99)*
Level of education: high	9.40 (2.55–34.67)**
(Still) working	4.02 (1.42–11.36)**
Retired	0.37 (0.16–0.83)*

**Multivariate**	

Age	0.90 (0.82–1.00)*
Married or living together with a partner	0.14 (0.04–0.48)**
Level of education: high	14.41 (3.27–63.56)**
(Still) working	8.88 (1.84–43.00)**

CI: Confidence Interval.

* *P *≤ 0.05; **P ≤ 0.01

#### Motives and barriers for participation

**Figure 2:**
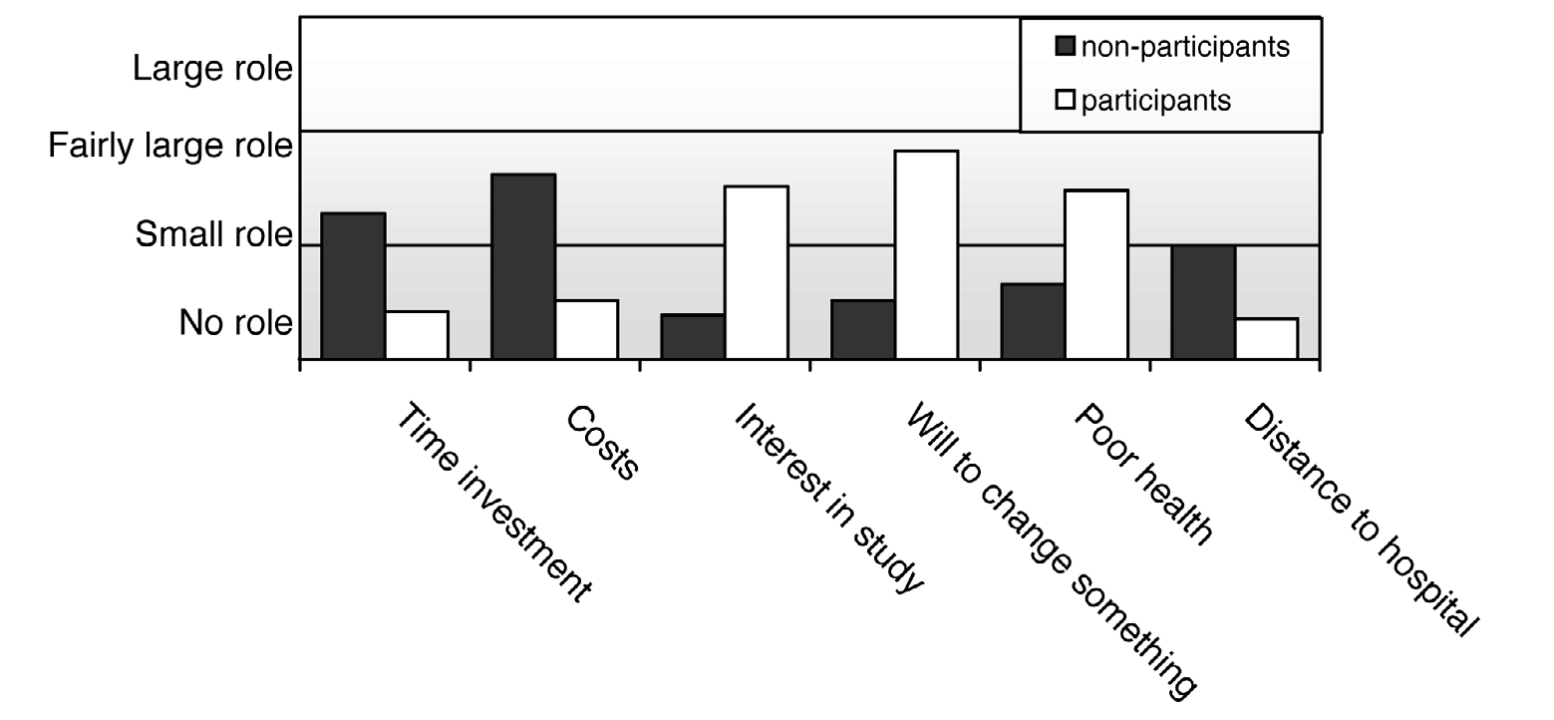
The role of determinants in the decision (not) to participate in the ALANT Study, reported on a four-item Likert scale by (non-)participants in a CVD lifestyle intervention trial.

Willingness to change lifestyle, perceived poor health, and interest in the study were reported as motives underlying the decision to participate (Figure 2).

The main reason for participation derived from the answers to the open question was: 'to improve my health' (33 participants). Altruistic reasons for participation were also mentioned, such as 'to help science' (8 participants). Non-participants stated that time investment (17 participants) and financial arguments (16 participants) played an important role in their decision to decline participation, and these were also the main reasons for non-participation derived from the answers to the open question.

The greatest motivational differences between participants and non-participants were the willingness to change and the level of interest in the study (Figure 2). Distance to the hospital (where the measurements took place) was also perceived as an important barrier for participation, even though the mean distance as measured from the Zip code of the patient's home address was similar for both groups.

## Discussion

The results of this study showed that the typical participant in a lifestyle intervention study for patients with stable cardiovascular disease was working, highly educated, younger, single patient. Participants consented in order to change their lifestyle, and/or because they wanted to improve their health. The main reasons for non-participation were financial arguments and time investment. The biggest motivational differences between participants and non-participants were willingness to change their lifestyle and interest in the study.

The observed demographical differences between participants and non-participants correspond with the findings of an earlier study of non-participation in cardiac rehabilitation [9] and a Dutch cardiovascular risk survey [19]. The motives underlying the decision to participate are also largely in line with the findings of an earlier study [7]. The statement made by Gates et al. [20] can be confirmed by our results: "Any cost (...) that patients perceive that they may incur if they participate is likely to affect their decisions about joining a trial".

The perception of poor health was more often a reason to participate than to decline; this is in contrast with previous reports in the literature [5,6]. Declining participation because of poor health was, in fact, the patient's subjective perception, because all the patients who were invited were assessed by their cardiologist as being healthy enough to participate. Although the distance to the hospital did not differ significantly between the two groups, non-participants experienced this distance as a barrier. Travel time and travel costs were not measured, but public transport facilities within the study area are good.

While non-participants were more often not employed, and probably had more free time, the time investment required for participation was a frequently reported barrier.

A few methodological issues need to be discussed. Firstly, non-response under the non-participants was relatively low (23%), so the absence of these pertinent non-respondents is not likely to bias our profile of the typical non-participant. Secondly, due to the relatively small sample size, not all elevated ORs are significant.

Thirdly, BMI was calculated with self-reported weight and height, which are known to be under-reported and over-reported, respectively, in epidemiologic studies [21]. When we compared the self-reported BMI of the participants with their BMI measured by the physician, we found a reported under-estimation with a mean difference of 0.7 kg/m^2^. However, this is not likely to bias the findings of BMI group-comparisons, since BMI was self-reported in both groups. Fourthly, we did not measure the enthusiasm and power of persuasion of the recruiters, which might be closely related to stimulation of a patient's interest [22]. However, we did measure lack of interest in the study, and this appeared to be a reason for non-participation in seven cases. Fifthly, most studies do not require payment from patients for the intervention. Therefore, the results have to be interpreted with caution with regard to comparison with other studies, and generalizability of the results. Furthermore, because study participation implied possible costs, it is plausible that patients with a low socio-economic status were less likely to participate. Unfortunately, we were unable to evaluate the exact socio-economic influences, because data on type of job or level of income were not collected. Based on the data concerning level of education there is reason to believe that socio-economic class is of influence. This is reinforced by the identified effect of the variable 'still working'.

## Conclusion

The differences between participants and non-participants are mainly based on demographic and motivational factors. Undoubtedly, the lifestyle intervention that was offered does not meet the needs of certain sub-groups. Further in-depth qualitative research could be beneficial in identifying the best way to promote a change in lifestyle in these groups. An individually tailored lifestyle program might increase the willingness of patients to participate.

The most important barriers for participation are costs and time investment. Although a lifestyle intervention is often focused on people with a lower socio-economic status, the financial burden of participation is likely to increase non-participation, especially in this sub-group. It is therefore recommended that participation should be stimulated financially, or at least be free of charge.

## Competing interests

The author(s) declare that they have no competing interests.

## Authors' contributions

JL wrote the initial draft and performed the analyses. WIJ, MvT, IMH and JCS participated in the design and co-ordination, and helped to draft the manuscript. JAR and AvR were involved in the data-collection and interpretation of the study results. The final version of the manuscript has been approved by all authors.

## Pre-publication history

The pre-publication history for this paper can be accessed here:


